# The impact of early diagnosis on the prognosis of extranodal NK/T-cell lymphoma with massive lung involvement: a case report

**DOI:** 10.1186/s12890-019-0815-9

**Published:** 2019-02-21

**Authors:** Tomohiro Yabushita, Satoshi Yoshioka, Takeru Furumiya, Momoko Nakamura, Daisuke Yamashita, Yukihiro Imai, Takayuki Ishikawa

**Affiliations:** 1 Department of Hematology, Kobe City Medical Center General Hospital, 2-1-1, Minatojima-Minamimachi, Chuo-ku, Kobe, 650-0047 Japan; 2 Department of Pathology, Kobe City Medical Center General Hospital, 2-1-1, Minatojima-Minamimachi, Chuo-ku, Kobe, 650-0047 Japan

**Keywords:** Lymphoma, Lung, Extranodal NK/T-cell lymphoma, Epstein-Barr virus

## Abstract

**Background:**

Pulmonary non-Hodgkin lymphoma (NHL) is rare. The most frequent subtype of pulmonary NHL is low-grade B-cell lymphoma, such as lymphoma of mucosa-associated lymphoma tissue. Extranodal natural killer cell/T-cell lymphoma, nasal type (ENKL) is characterized by predominant extranodal involvement and association with Epstein-Barr virus (EBV). ENKL with massive lung involvement has been infrequently reported, and its prognosis is extremely poor.

**Case presentation:**

A 20-year-old Japanese man presented with intermittent fever lasting for 2 months. Radiological imaging demonstrated multiple nodules of uneven shape and size in both lungs. Video-assisted thoracic surgical lung biopsy showed abnormal lymphocyte infiltration, which was positive for CD3, CD56, and perforin. In situ hybridization for EBV-encoded RNA was positive. From these findings, he was diagnosed with ENKL with lung involvement. The patient was successfully treated with intensive combinational chemotherapy followed by allogeneic cord blood transplantation. He has been alive with continuous complete remission for 1 year after diagnosis.

**Conclusions:**

Although ENKL involving the lung has been reported to have dismal outcomes, our patient showed long-term survival after intensive chemotherapy and up-front allogeneic hematopoietic transplantation. The present case highlights the importance of early diagnosis as well as allogeneic transplantation.

## Background

Pulmonary non-Hodgkin lymphoma (NHL) is a rare disease representing approximately 1% of all NHLs and 0.5–1.0% of all pulmonary malignancies [1]. The majority of pulmonary NHLs are of mature B-cell lineage and most are lymphomas of mucosa-associated lymphoma tissue, characterized by an indolent clinical course and good treatment response. In contrast, mature T-cell or natural killer (NK) cell lymphomas involving the lung are less common, and usually have an aggressive clinical course [2].

Extranodal NK/T-cell lymphoma, nasal type (ENKL) is a subtype associated with Epstein-Barr virus (EBV) infection and commonly involves the upper respiratory tract, such as the nasal cavity, nasopharynx, and paranasal sinuses [3]. In previous reports, approximately 14–23% of ENKLs occurred in extranasal sites: the skin, soft tissue, gastrointestinal tract, and testis [4–7]. Pulmonary involvement of ENKL (pulmonary ENKL) is extremely rare. Here, we report a young Japanese man with pulmonary ENKL who was successfully treated with intensive combination chemotherapy and cord blood transplantation (CBT).

## Case presentation

**Fig. 1:**
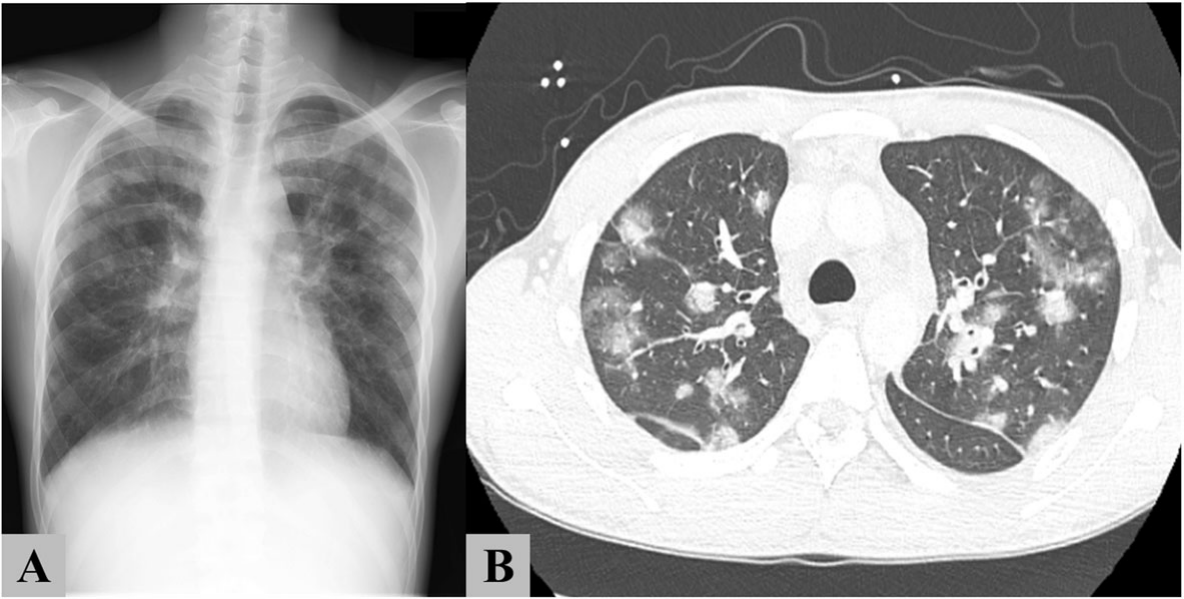
Radiological imaging on admission. Chest computed tomography revealed multiple nodules in both lungs that showed diffusely mixed with consolidation and ground-glass opacity pattern. (**a**; coronal section, **b**; horizontal section)

**Fig. 2:**
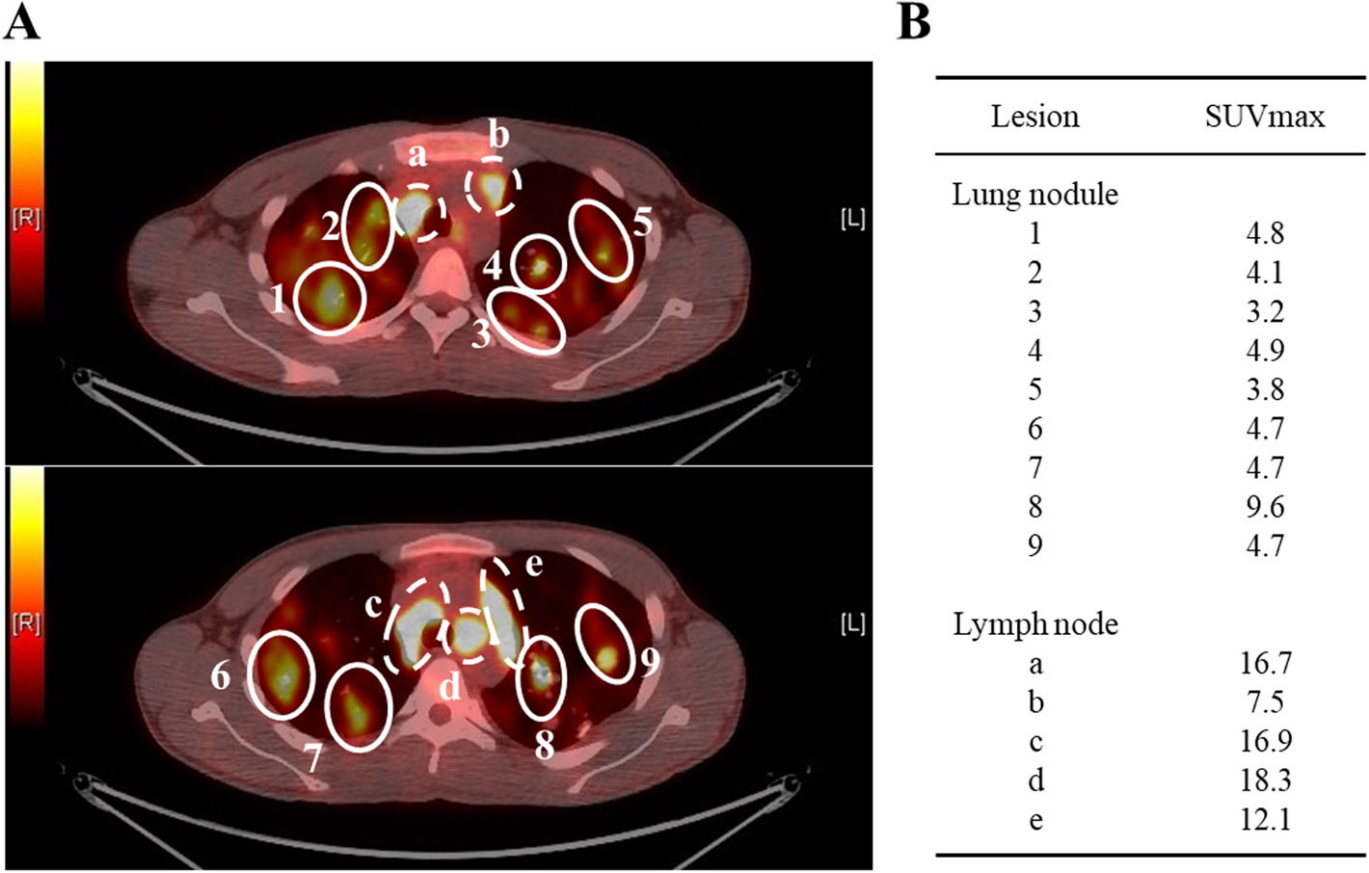
The axial view of FDG-PET/CT scan on diagnosis. (A) PET/CT scan shows intense FDG uptake in bilateral lung masses and the mediastinal and hilar lymph nodes. Each lung nodule is surrounded by a solid-line circle (1–9). Each mediastinal lymph node is surrounded by a dotted line circle (a-e). (B) The maximum standardized uptake values (SUVmax) of the lesions are shown here

A 20-year-old Japanese man presented with a 2-month history of fever, night sweats, and mild weight loss. He had no rash or palpable peripheral lymphadenopathy. Laboratory tests revealed anemia (hemoglobin, 9.5 g/dL), leukopenia (white blood cell, 2.4 × 10^9^/L), and elevated lactate dehydrogenase (LDH, 1175 U/L; normal range 120–250 U/L). The EBV-DNA level was extremely high in the whole blood (4.0 × 10^6^ copies/mL). Abnormal cells were not detected in the peripheral blood (PB). Other laboratory data are shown in Table 1. Chest radiography showed bilateral pulmonary lesions predominantly in the upper lung. Computed tomography (CT) showed multiple nodules diffusely mixed with consolidation and ground-glass opacity pattern in both lungs (Fig. 1). Enlargement of the mediastinal and hilar lymph nodes and hepatosplenomegaly were also observed. Positron emission tomography (PET)/CT showed abnormal uptake of 18-fluorodeoxyglucose (FDG) in multiple lung lesions, as well as the mediastinal and hilar lymph nodes, bilateral humeral bones, lumbar spine, liver, and spleen. The maximum standardized uptake value (SUVmax) of lung nodules (median, 4.7; range, 3.2–9.6) was lower than that of hilar lymph nodes (median, 16.7; range, 7.5–18.3) (Fig. 2).

**Table 1:** Laboratory Data at the time of his admission

Complete blood counts		Biochemistry		Viral and Fungal Markers	
WBC	2400 /μL	TP	6.1 g/dL	HBs Ag	0.0IIU/mL
Neut.	63.0%	Alb	3.4 g/dL	HBs Ab	0.0 mIU/mL
Lymph.	22.0%	AST	59 U/L	IgM-HBcAb	0.1 S/CO
Mono.	15.0%	ALT	58 U/L	HCVAb	0.1 S/CO
Abnormal lymph.	0.0%	LDH	1181 U/L	HIV1/2Ab	0.1 S/CO
RBC	322 × 10^4^ /μL	T-bil	0.6 mg/dL	EBV-VCA IgM	(−)
Hb	10.2 g/dL	BUN	9.6 mg/dL	EBV-VCA IgG	(+)
Plt	14.1 × 10^4^ /μL	Cre	0.71 mg/dL	EBV-EBNA	(+)
Coagulation tests		Na	137 mEq/L	EBV-DNA	4,000,000 copy/mL
PT-INR	1.21	K	3.8 mEq/L		
APTT	45.8 s	CRP	1.01 mg/dL		
Fib	523 mg/dL	sIL-2R	4863 U/mL		
D-dimer	7.86 μ/mL	ferritin	2926 ng/mL		

*WBC* white blood cells, *Neut* neutrophils, *Lymph* lymphocytes, *Mono* monocytes, *Abnormal lymph* Abnormal lymphocytes, *RBC* Red blood cells, *Hb* hemoglobin, *Plt* platelet, *PT* prothrombin time, *APTT* activated partial thromboplastin time, *Fib* fibrinogen, *TP* total protein, *Alb* albumin, *AST* asparate aminotranferase, *ALT* alanine aminotransferase, *LDH* lactate dehydrogenase, *T-bil* total bilirubin, *BUN* blood urea nitrogen, *Cre* creatinine, *CRP* C-reactive protein, *sIL-2R* soluble IL-2 receptor, *Ab* antibody, *Ag* antigen, *HB* hepatitis B virus, *HCV* hepatitis C virus, *HIV* human immunodeficiency virus, *EBV* Epstein-Barr virus VCA; viral capsid antigen, *EBNA* EBV nuclear antigen

**Fig. 3.**
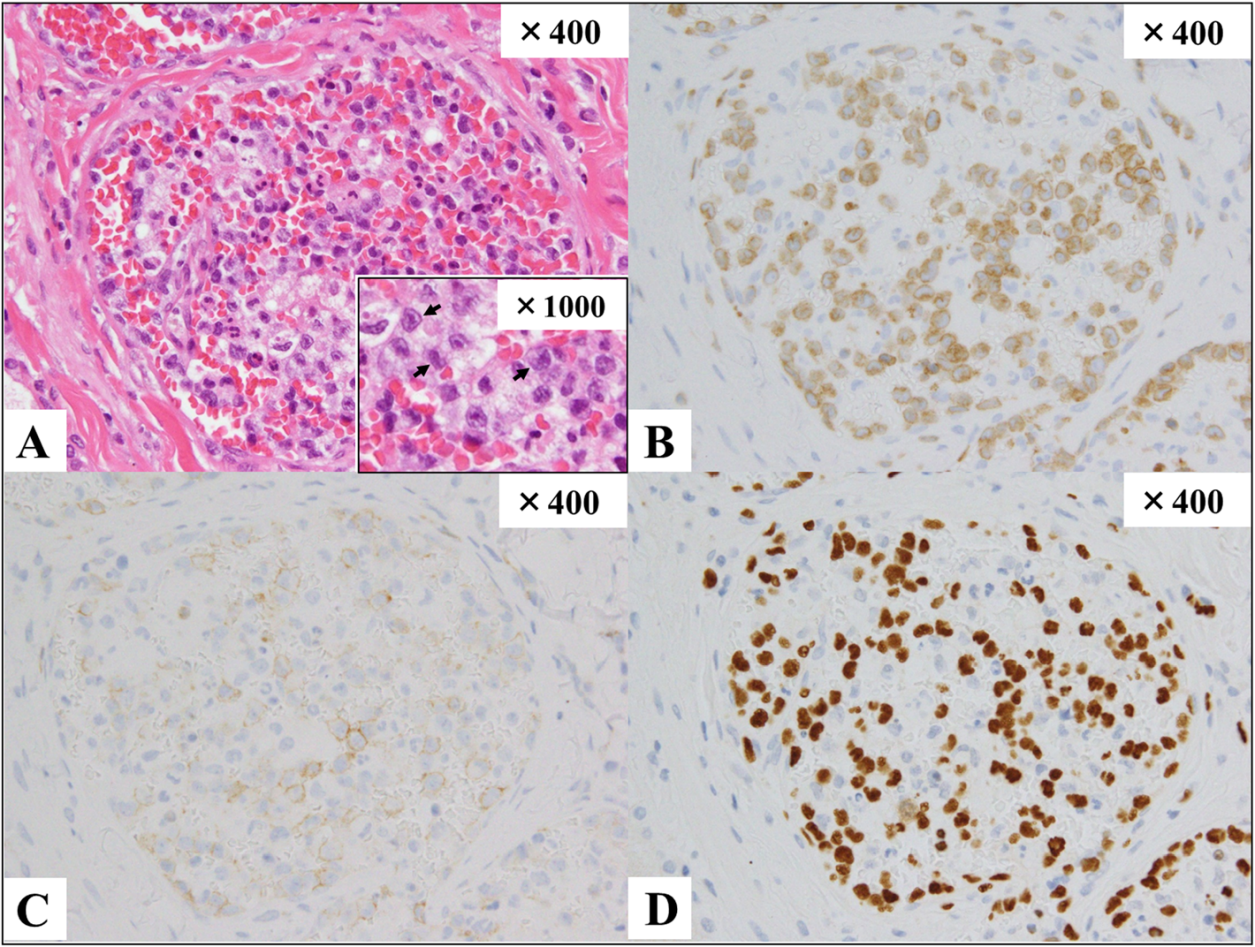
Pathological findings of the lung biopsy. Atypical large-sized lymphoid cells filled several distended vessels beneath the pleura and in the pulmonary parenchyma (hematoxylin and eosin; × 400 (A)). The neoplastic cells had irregular nuclear contours, prominent nucleoli, and narrow cytoplasm (black arrow), described in a partially expanded image on the lower right field. Abnormal mitosis was prominent in the cells. The moderate inflammatory response caused mainly by plasma cells was seen in perivascular areas, but no fibrin thrombi or necrosis was observed. The alveolar epithelial cells showed reactive nuclear enlargement. The tumor cells in pulmonary arterioles were positive for cytoplasmic CD3 (B), CD56 (C), perforin, and Ki-96 (not shown). In situ hybridization analysis for Epstein-Barr virus-encoded messenger RNA was positive (D).

Video-assisted thoracoscopic surgical lung biopsy (VATS) was performed the next day. Visual inspection revealed dark purple patchy lesions on the whole lung surface, and specimens were obtained from the left S6 and S1 + 2. Pathological examination showed large-sized atypical cell infiltration localized mainly in the lumina and perivascular areas of the distended vessels beneath the pleura and in the pulmonary parenchyma (Fig. 3). These abnormal cells had irregularly contoured nuclei, prominent nucleoli, and narrow cytoplasm. The tumor cells were positive for cytoplasmic CD3ε, CD56, and perforin, and negative for cytokeratin, CD20, and CD30 by immunohistochemistry. EBV infection was detected by in situ hybridization analysis for EBV-encoded RNA. The Ki-67 proliferation index showed 70–80% nuclear staining. T-cell receptor γ chain rearrangement was not proven, which was consistent with an NK-cell origin. Based on these findings, he was diagnosed with ENKL (Stage IV). The bone marrow was normocellular with an increase in activated histiocytes containing engulfed red blood cells, nuclear debris, and platelets, but no neoplastic cell infiltration was observed. This finding was compatible with hemophagocytic lymphohistiocytosis (HLH).

One week after admission, he received combination SMILE regimen chemotherapy (dexamethasone, methotrexate, ifosfamide, l-asparaginase, and etoposide). After two cycles of SMILE, complete remission (CR) was confirmed by FDG-PET/CT, and EBV-DNA became undetectable in the PB. Subsequently, he received a single unit CBT after myeloablative conditioning of cyclophosphamide (120 mg/kg) plus 12 Gy total-body irradiation. Graft-versus-host disease (GVHD) prophylaxis consisted of tacrolimus and mycophenolate mofetil. On day 16, he achieved successful neutrophil engraftment. On day 21, he developed biopsy-proven gastro-intestinal acute GVHD (grade II), which gradually improved with prednisolone at doses of 0.5 mg/kg daily. On day 88, he was discharged in complete remission with no other major complication. Four months later, he developed mild cutaneous chronic GVHD limited to the head and neck, which could be managed with topical steroids. He has been alive with continuous CR for 1 year after diagnosis.

## Discussions and conclusions

ENKL is a rare type of lymphoma associated with EBV infection that most commonly involves the upper aerodigestive mucosa and skin. Although localized ENKL has a better prognosis when treated with concurrent chemoradiotherapy [8], advanced ENKL often displays a fulminant clinical course with very poor prognosis. Therefore, especially in advanced ENKL, a delay in diagnosis could provoke multiple organ dysfunction and make treatment difficult. In our case, surgical lung biopsy contributed to the rapid and precise diagnosis of ENKL, which resulted in maximal therapeutic efficacy.

ENKL involving the lung is rare, and only 14 cases of ENKL with massive pulmonary involvement have been reported in the English literature [2, 9–16]. The diagnosis of pulmonary ENKL is often difficult because of the nonspecific clinical symptoms and radiological findings. In previously published reports, the major initial symptoms of this disorder were fever (*n* = 12) and cough (*n* = 10). Moreover, the common CT findings were multiple nodules in both lungs (*n* = 9), consolidation (*n* = 4), bronchial or mediastinal involvement (*n* = 3), pleural effusion (*n* = 3), and a single lesion in one lung (*n* = 2). Although laboratory data were not available in all cases (*n* = 6), pancytopenia (*n* = 4) and elevated LDH (median, 498 U/L; range 135–1192 IU/L) were commonly observed.

Lung biopsy is required for definite diagnosis of ENKL mainly involving the lungs. Eleven of the 14 patients were diagnosed using lung biopsy, including transbronchial lung biopsy (TBLB; n = 2), CT-guided biopsy (*n* = 2), surgical biopsy (*n* = 1), and unspecified core biopsy (*n* = 6). The other 3 patients were diagnosed at autopsy despite repeated antemortem biopsies. Specimens obtained by TBLB or CT-guided biopsy are often inappropriate because of their small-sized sampling. In contrast, surgical biopsy enables a sufficient amount of specimen for precise diagnosis. Therefore, the surgical approach should be considered as a helpful diagnostic method for ENKL before rapid exacerbation, especially when ENKL is suspected on the basis of clinical features. In the present case, considering the complication of HLH and the highly elevated level of EBV-DNA, we included ENKL in the differential diagnosis list, and chose surgical biopsy as a diagnostic procedure before rapid exacerbation.

Some ENKL cases are complicated by lymphoma-associated HLH, which usually presents with persistent high fever, pancytopenia, hepatosplenomegaly, or elevated levels of soluble interleukin-2 receptor or ferritin. Approximately half of the cases of lymphoma-associated HLH have been reported to be correlated with EBV-associated lymphoid malignancies, such as ENKL [17]. In addition, it is well known that almost all ENKL cases show positive EBV-DNA in their PB, but not in the normal control. EBV-DNA detection using PB samples is very helpful for diagnosis.

The prognosis of advanced ENKL is very poor; overall survival (OS) at 5 years for advanced ENKL has been reported to be approximately 25% [8]. Pulmonary ENKL usually has a fatal outcome [18, 19]. Eight of the 14 patients described in the literature were treated with chemotherapy. One received autologous hematopoietic stem cell transplantation followed by high-dose chemotherapy. However, all died of disease progression regardless of intensive treatment. The median OS in 12 evaluable patients was 2 months (range, 1 week to 7 months). Recently, combinational chemotherapy containing L-asparaginase (the SMILE regimen) has been reported to be effective for ENKL compared to the conventional anthracycline-based regimen, such as CHOP (cyclophosphamide, doxorubicin, vincristine, and prednisone) [20]. In several guidelines, subsequent hematopoietic stem cell transplantation (HSCT) is recommended for patients with advanced ENKL achieving CR after induction chemotherapy because of its poor prognosis [21, 22]. We determined to perform allogeneic (allo-) HSCT for this patient after achieving CR with two courses of the SMILE regimen, because the extremely poor prognosis of pulmonary ENKL was estimated from the above literature review. Since neither related nor unrelated HLA-matched donor was found, we selected cord blood donor. He has been alive with continuous CR for 1 year after diagnosis. However, there is no clear evidence to determine whether autologous (auto-) or allo-HSCT is preferred for advanced ENKL. Yim et al. reported the outcome of upfront auto-HSCT for ENKL. Patients with advanced disease had significantly worse prognosis than those with limited disease: 3-year progression free survival (PFS), 40.1% versus 64.5%, *p* = 0.017; OS, 52.3% versus 67.6%, *p* = 0.048 [23]. In contrast, allo-HSCT for ENKL has been evaluated only by small retrospective studies: OS rates were 34–57% [24–26]. In a retrospective nationwide survey of auto-HSCT and allo-SCT for Japanese patients with ENKL, the 2-year OS in auto-HSCT group was superior compared to allo-SCT group: 69% versus 41%, *p* = 0.002. However, when adjusted by advanced stage, disease status, and performance status at transplant, there was no significant difference of 2-year OS between both transplant procedures, because the allo-HSCT group included more patients with advanced stage and refractory disease condition [27]. Allo-SCT could be one of the curative options for some patients with advanced ENKL. In general, the primary indication for allo-HSCT should be cautiously considered in each individual case.

In summary, we described a rare case of a pulmonary ENKL in a 20-year-old man who was successfully diagnosed using early surgical biopsy and treated with intensive multi-agent chemotherapy and up-front CBT, and he has survived more than 1 year with no relapse. In the present case, VATS was helpful for prompt diagnosis and successful treatment. Collection of sufficient specimen with any invasive procedure is crucial for diagnosis of pulmonary aggressive lymphoma like ENKL.

## Abbreviations

CBT: cord blood transplantation
CHOP: cyclophosphamide, doxorubicin, vincristine and prednisone
CR: complete response
CT: computed tomography
EBV: Epstein-Barr virus
ENKL: extranodal NK/T-cell lymphoma, nasal type
FDG: 18-fluorodeoxyglucose
HLH: hemophagocytic lymphohistiocytosis
LDH: lactate dehydrogenase
NHL: non-Hodgkin lymphoma
NK: natural killer
OS: overall survival
PB: peripheral blood
PET: positron emission tomography
SMILE: dexamethasone, methotrexate, ifosfamide, l-asparaginase, and etoposide
TBLB: transbronchial lung biopsy
VATS: video-assisted thoracoscopic surgical lung biopsy
